# Optical coherence tomography angiography suggests choriocapillaris perfusion deficit as etiology of acute macular neuroretinopathy

**DOI:** 10.1007/s00417-024-06436-7

**Published:** 2024-03-21

**Authors:** Claus von der Burchard, Arved Gruben, Johann Roider

**Affiliations:** Department of Ophthalmology, University Medical Center Schleswig-Holstein, Arnold-Heller-Str. 3, 24105 Kiel, Germany

**Keywords:** AMN, Acute macular neuroretinopathy, OCTA, Vessel area density

## Abstract

**Purpose:**

Acute macular neuroretinopathy (AMN) can cause sudden-onset and permanent scotoma in healthy young patients. Analysis of optical coherence tomography (OCT) and OCT angiography (OCTA) of AMN patients may provide insights into disease mechanism.

**Methods:**

We conducted a retrospective study of consecutive SARS-Cov-2-related AMN patients that presented in our clinic between Jan 1st, 2022, and April 30th, 2023, within 30 days of symptom onset. Retinal vessel area density (VAD) of AMN lesions in OCTA was quantified and compared to an adjacent tissue control (ATC). This quantification was performed for the superficial vascular plexus (SVP), the intermediate capillary plexus (ICP), the deep capillary plexus (DCP), the choriocapillaris (CC), and choroid. Furthermore, en face OCT images were analyzed.

**Results:**

Nine AMN patients were identified, 6 of these (4 female, 2 male, average age 25 years) fulfilled the inclusion criteria and were included into this study. Average time from symptom onset to OCTA was 14.3 days. No VAD differences between AMN and adjacent tissue were found in either retinal layer (SVP, ICP, DCP). In contrast, VAD in CC was reduced by 27% against the ATC (*p* = 0.007) and choroidal VAD was reduced by 41% (*p* = 0.017). Further analysis of en face OCT could show that the pathognomonic infrared hyporeflectivity in AMN is caused by photoreceptor alterations rather than changes in the inner retinal layers.

**Conclusions:**

Our data suggests that a perfusion deficit in the choroidal layers is responsible for AMN rather than in the DCP, which is the predominant hypothesis in current literature.

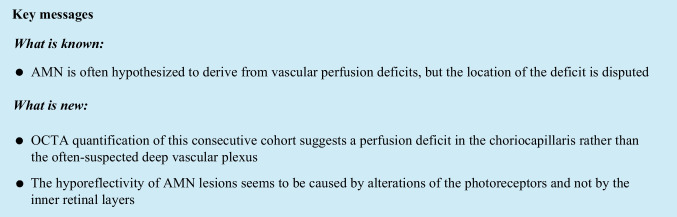



## Introduction

Acute macular neuroretinpathy (AMN) is a retinal finding first described in 1975 by Bos and Deutman [1] that is defined by the development of sudden-onset distinct parafoveal scotomas with associated hyporeflectivity in infrared (IR) confocal scanning laser ophthalmoscopy (cSLO). The disease usually affects younger subjects (20–30 years old) with a strong female predominance (around 85%) [2]. In most cases, association with concurrent viral infections [2] is found, but also vaccination-associated (e.g. [3–6]) and idiopathic occurrences were reported. Since the beginning of the SARS-CoV-2 pandemic, a strong rise in incident has been reported [7]. Also, a strong association to the use of oral contraceptives was found (35% in one review [2]).

The disease can present with one or multiple scotomas in one or both eyes. The etiology is still unclear and widely discussed in literature. Initially, most authors presumed a capillary occlusion of the deep retinal capillary plexus (DCP) [3, 8–13]. This hypothesis lends itself as plausible because both the distinct hyporeflectivity in infrared cSLO and the distinct hyperreflectivity of localized layers in optical coherence tomography (OCT) resemble cases of retinal artery occlusion, except for the circumscribed OCT findings in AMN affecting only some but not all inner retinal layers.

To make things more complicated, however, AMN has been subdivided into two or even three groups with distinct layer affection: AMN type 1, also called paracentral acute middle maculopathy (PAMM) [14], shows OCT alterations superior to the outer plexiform layer (OPL), whereas the more-often [15], “classic” AMN type 2, also called acute macular outer retinopathy [16], affects the outer retina below the OPL. Hufendiek et al. [15] first described AMN type 3, which is characterized with more severe visual impairment, subretinal fluid, and atypical infrared cSLO hyper- instead of hyporeflectivity. The following analysis only analyzes cases of “classic” AMN type 2.

OCT angiography (OCTA) allows for a detailed analysis of retinal vasculature and identification of which plexus might be affected. Therefore, different groups have analyzed OCTA in AMN, but mostly in small case series. However, the findings of the studies have varied widely, and while some authors found evidence in favor of the suspected DCP origin, others have found contradicting data.

This paper therefore applies OCTA analysis to a consecutive case series of AMN patients for an unbiased analysis. It also provides en face OCT analysis to back the findings.

## Methods

### Patient selection

We retrospectively analyzed all patients in our clinic that were diagnosed with AMN during an acute SARS-CoV-2 infection between Jan 1st, 2022, and April 30th, 2023. Inclusion criteria were all patients with the diagnosis of an AMN. The type of AMN was defined as proposed by Sarraf [14] and Hufendiek [15]: Briefly, both type 1 and type 2 AMN are defined by demarked hyporeflective infrared lesions with corresponding OCT alterations, which in type 1 lie above the OPL and in type 2 below the OPL. Type 3 instead shows hyperreflective infrared lesions with subretinal fluid in OCT. We excluded all patients where the initial presentation was more than 30 days after symptom onset or with bilateral disease.

The study was approved by the Institutional Review Board under the permission no. D608/23 (Ethics Committee, Kiel University).

### OCTA acquisition and quantification

All OCTA images were acquired with a Spectralis HRA + OCT2, Heidelberg Engineering, Heidelberg, Germany, with 6 µm interscan distance. For quantification, all predefined slabs (superficial vascular plexus (SVP), intermediate capillary plexus (ICP), deep capillary plexus (DCP), choriocapillaris (CC), and choroid) and the corresponding infrared image were exported from the proprietary image viewer software as images in PNG file format. A previous manual examination had revealed that the automated OCT segmentation for slab creation was without critical errors in all cases.

**Fig. 1:**
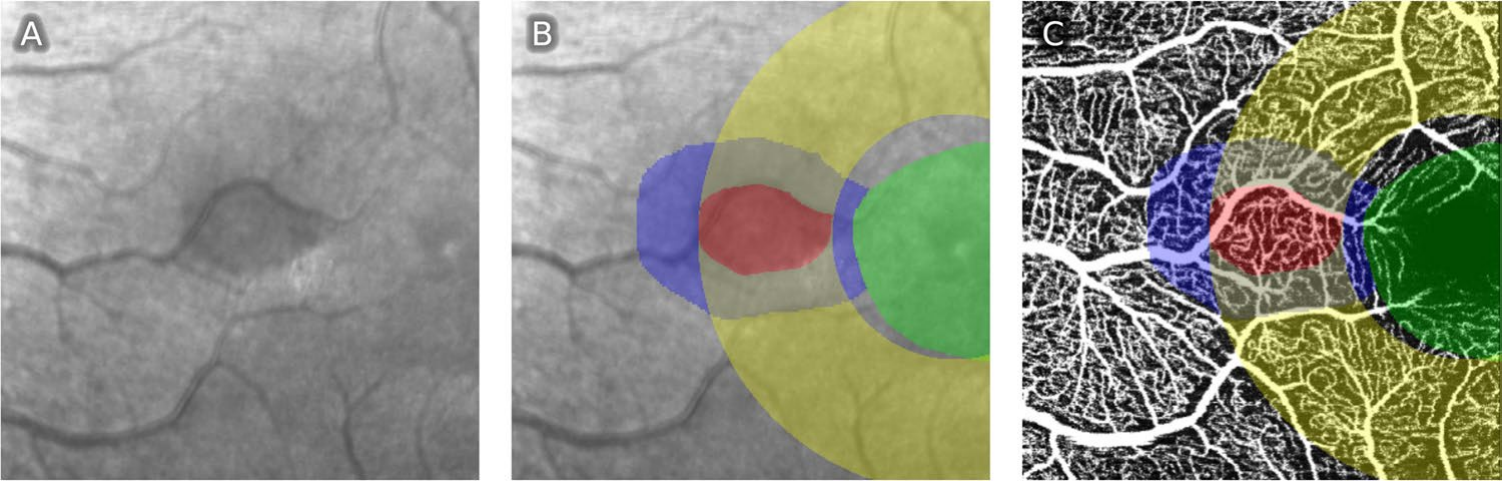
Analyzed segmentations in one sample patient. **A** Raw IR image. **B** Segmentations on IR image: AMN (red), FAZ (green), adjacent tissue control (blue), ring segment (yellow). **C** Same segmentations shown on SVP

A custom-written python software (“Open OCTA Analyzer”) was then used to further process, quantify, and analyze the OCTA data. First, the images were converted to Nifti file format. The infrared scan was then segmented in ITK-Snap [17], where the AMN lesion (Fig. 1, red segmentation) and the foveolar avascular zone (FAZ; Fig. 1, green segmentation) were segmented. The script then automatically calculated two regions to compare the vessel area density to:

- First, the adjacent tissue control (ATC; Fig. 1, blue segmentation), which was defined as the areas surrounding the AMN. For this, the AMN segmentation was doubled in size with the same midpoint, then the original lesion was excluded, so that only the tissue directly adjacent to the lesion remained. If parts of ATC lay within the FAZ, these parts were also excluded.
- Second, a ring segment (RS; Fig. 1, yellow segmentation) was defined by two concentric circles around the fovea so that both circles were tangents of the AMN segmentation; the AMN segmentation was again excluded from this segment. This was done in the understanding that because all parts of the ring segment had equal distance to the fovea and because of the vascular architecture of the foveal region, homogenous vascular density might be suspected in this region.

Both comparators ATC and RS were designed before conduct of statistical analysis. The vessel area density (VAD) [18] was calculated similar as described before by different authors [19, 20]. We used an Otsu thresholding algorithm [21] to separate perfused (above threshold) from non-perfused (below threshold) tissue. We found that additionally applying a Frangi vesselness filter [22] as described by Hosari [20] did not substantially alter measurements for retinal vasculature analysis, but disallowed for measurements of choriocapillaris and choroid vessel area density; therefore, we only used the Otsu thresholding. Afterwards, the number of above-threshold pixels in the different segmented areas was divided by the overall pixel sum to find the average VAD. For quantification of the SVP, a Frangi vesselness filter configured to detect only large vessels was used to exclude these vessels from analysis. For significance testing, a paired samples *t*-test with Bonferroni correction was used. Statistical significance was defined as *p* < 0.05.

The entire python software package is published open-source and can be used freely for reproduction studies or other OCTA analysis under https://github.com/clausvdb/OpenOCTAAnalyzer.

### En face OCT analysis

For en face OCT analysis, en face reconstructions of a densely sampled OCT volume scan over the lesion was exported in a standardized manner at the junction of the outer plexiform layer (OPL) to the Henle fibers and at the ellipsoid zone (EZ). The corresponding IR image was also exported. In all images, the AMN lesion area was then segmented with ITK-Snap. The overlapping of the EZ and OPL layer with the IR lesion was determined by a Sørensen–Dice score [23].

## Results

### Patient demography

Within the inclusion period, we identified 9 SARS-Cov-2-related AMN patients (2 male, 7 female) with an average age of 26.1 years (range 19–36 years). All patients reported a sudden onset of a paracentral scotoma and showed clearly demarked alterations both in the infrared and OCT images that were consistent with previous reports of AMN. All cases could be classified as type 2 AMN, and all AMN lesion laid outside of the FAZ. All female patients used oral contraception (2 estrogen-free “mini pills,” rest unknown). Two female patients presented with bilateral, multifocal lesions, whereas the rest presented with a unilateral, focal lesion (4 left eyes, 3 right eyes).

Time from symptom onset to first OCTA acquisition ranged from 0 to 100 days. We only included patients into further analysis where the first OCTA image was acquired within 30 days of symptom onset and with unilateral, focal lesions (6 patients, 4 female). Table 1 shows an overview of the patient’s demographic.

**Table 1:** Patient demographics. *Exclusion criteria: time delay from symptom onset to OCTA > 30 days (patient 7 and 9); bilateral, multifocal lesions (patient 8 and 9). BCVA, best-corrected visual acuity. In bilateral disease, the visual acuity is noted as right eye/left eye

Patient No	Gender	Affected eye	BCVA (decimal)	Age	Average age	Time delay symptoms-OCTA (days)	Average delay
Included in analysis							
1	F	Left	1.0	19	25	13	14
2	F	Right	1.0	20		27	
3	F	Left	1.0	22		7	
4	F	Left	1.25	25		20	
5	M	Right	1.0	28		17	
6	M	Left	1.25	36		2	
Excluded from analysis*							
7	F	Right	1.25	25	26.7	100	60
8	F	Both	1.0/1.0	27		1	
9	F	Both	1.2/1.2	28		78	

### OCTA vessel area density

**Fig. 2:**
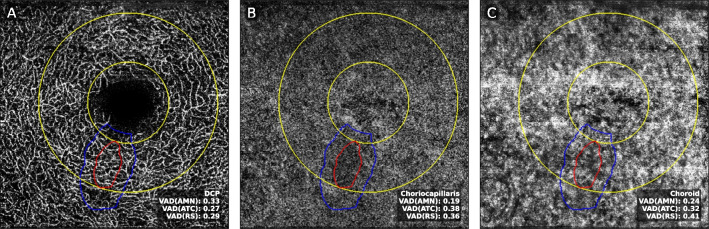
Representative vessel area density VAD calculation of an AMN patient. **A** Deep capillary plexus (DCP). **B** Choriocapillaris (CC). **C** Choroid slab. VAD vessel area density. AMN acute macular neuroretinopathy. ATC adjacent tissue control. RS ring segment

For all images, vessel area density (VAD) was calculated as described in the “Methods” section. Figure 2 shows a representative analysis of the DCP, CC, and choroid slab and the respective vessel area density measurements.

**Fig. 3:**
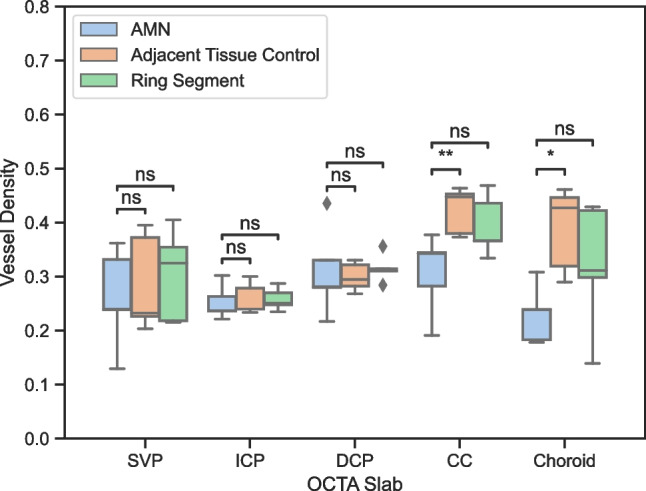
Statistical analysis of vessel densities of all patients for different OCTA slabs. SVP superficial vascular plexus, ICP intermediate capillary plexus, DCP deep capillary plexus, CC choriocapillaris. * *p* < 0.05; ** *p* < 0.01

Overall, it could be shown that vessel area density in the AMN area was not significantly different to the adjacent tissue control or the ring segment in any retinal vascular layer, i.e., SCP (*p* = 0.70 for ATC; *p* = 0.72 for RS), ICP (*p* = 0.43/*p* = 1.0), or DCP (*p* = 1.0/*p* = 1.0). In contrast, it could be shown that VAD was reduced in both choroidal layers. On average, CC vessel area density (VAD = 0.31) was reduced by 27% against the ATC (*p* = 0.007) and 22% against the RS (*p* = 0.09). The choroidal vessel area density (VAD = 0.23) was reduced by 41% against the ATC (*p* = 0.017) and 28% against the RS (*p* = 0.27). Figure 3 shows a box plot analysis of these findings.

### En face analysis

**Fig. 4:**
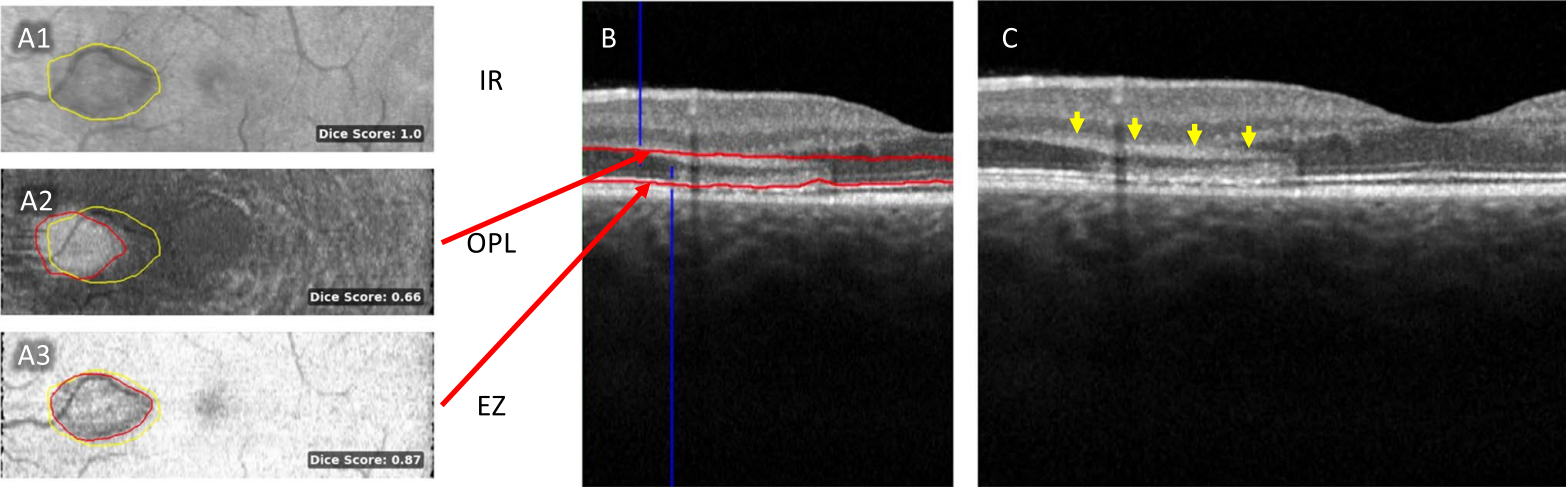
Dice similarities between en face images. **A1** Infrared image. **A2/A3** En face reconstructions of the OPL and EZ layer. Yellow circle: segmentation of the IR hyporeflectivity. Red circle: segmentation of the alterations in the respective en face OCT scan. **B/C** Corresponding OCT images with and without depth information of en face reconstructions. It can be clearly seen how the infrared lesion correlates well between the infrared image and the EZ layer, whereas the Henle fibers (yellow arrowheads) diverge nasally. The dice score is therefore much higher for the EZ layer (0.87 in this example) than for the OPL layer (0.66). IR infrared, OPL outer plexiform layer, EZ ellipsoid zone

For all patients, we checked if the OCT alterations were clearly distinguishable in en face OCT both at the EZ layer and at the junction of the outer plexiform layer (OPL) to the inner nuclear layer (INL). In 4 patients, these conditions were given. In these patients, a distinct swelling of the Henle fiber layer was visible (Fig. 4, yellow arrowheads). As can be seen in Fig. 4C, often these swellings of the Henle fibers tilted away from the fovea due to the foveal anatomy.

This allowed for comparison between the location of OCT alterations and IR hyporeflectivity. A Dice score was used to determine the amount of overlap between the IR alterations and the alterations in the en face reconstruction of the ellipsoid zone (EZ) and outer plexiform layer (OPL), respectively. As shown exemplarily for one patient in Fig. 4A, analysis of all patients showed that the Dice score was much higher for the EZ-IR overlap (Dice score = 0.89 ± 0.03) than for the OPL-IR overlap (Dice score = 0.65 ± 0.03). Using a paired *t*-test, we could demonstrate that this elevation in the Dice score for the EZ-IR overlap in comparison to the OPL-IR overlap was statistically significant (*p* = 0.00078). This suggests that the IR hyporeflectivity is caused by alterations on the photoreceptor level and not by alterations in the OPL.

## Discussion

### Findings of the study and dispute over AMN pathogenesis

In our study, we only found significant perfusion deficits in the choriocapillaris and choroid layer, but no changes in the blood perfusion of the retinal layers. Since this contradicts earlier findings which suspect a DCP origin, further insight into the different plexuses and their potential role in AMN is needed.

Although the precise anatomy of retinal capillary plexuses and their watersheds are under debate, the DCP is generally considered to supply the inner nuclear layer (INL) and outer plexiform layer (OPL). Any DCP circulation deficit, therefore, should rather affect these inner retinal layers as seen in PAMM, and indeed, this is the suspected pathomechanism for PAMM [24, 25]. Given this, it remains unclear how a perfusion deficit in the DCP should be able to cause both PAMM and AMN, two entities that not only affect different retinal layers, but also have different epidemiologies [26].

The hallmark of AMN type 2, in contrast, is less alterations in the inner retinal layers, but rather a disruption of the ellipsoid zone. Adaptive optics investigations have confirmed that structural alterations happen in the photoreceptor layer [27, 28], and so does our en face OCT analysis. Because the photoreceptors are nourished by the underlying choriocapillaris, no microvascular perfusion in the retina should cause these changes. If the underlying cause was a retinal perfusion deficit in the DCP, then the photoreceptor alterations could only be explained by retrograde transneuronal degeneration, i.e., propagation of an inner retinal defect onto the photoreceptors. However, no such behavior can be seen in retinal artery occlusion, and also, the early onset of alterations within hours is implausible for this theory.

Lately, a shift in reporting has occurred. A focal perfusion deficit in the choroidal layers could in theory much better explain the isolated alterations on the photoreceptor layer in AMN. As Bottin [29] and Munk [30] pointed out, the changes that are often attributed to the outer plexiform layer (i.e., domain of the DCP blood supply) are much rather swellings of the Henle fiber layer (HFL). The Henle fibers are the axons of the photoreceptors that connect the photoreceptor cells to the outer plexiform layer and are thus dependent on choroid blood flow. In AMN occurrences near the foveal depression, the sidewards tilt of these fibers can be visualized, as in Fig. 4C (yellow arrowheads). Thanos et al. [31] were the first to report on choroidal perfusion deficits that could be observed in OCTA analysis of AMN cases. This finding was later backed by other investigators [32, 33], although these findings did not go undisputed [9]. This theory much better explains the structural alterations that are mainly in the photoreceptors. Duan et al. proposed the double watershed theory, which presumes a concurrent relative perfusion deficit both from the DCP and the choroidal vasculature [34] that ultimately results in AMN lesions.

### Interpretation of contradicting results from different studies

The contradicting findings of the authors suspecting a choroidal perfusion deficit versus the authors that have found DCP perfusion deficits within the same imaging modality (OCTA) [3, 8–13] remain at least partially unexplainable. One major factor, however, could be that on top of different OCTA devices with different wavelengths and image output, no standardized measurements of OCTA perfusion exist. Probably, the most important issue is the non-availability of standardized OCTA quantification software. Even though different authors have used similar principles, most of the used OCTA analysis software remains unavailable to other research groups, which hinders reproduction studies. We therefore chose to release our quantification software open source, so that future authors can use the same methodology for reproduction studies or other scientific questions.

Another factor is that different authors have used different controls (from surrounding area to unaffected partner eye to healthy controls). We specifically chose our control parameters within the same eye of the same patient to make the findings less prone to intra- and interindividual alterations of OCTA. VAD is known to vary not only in diseased but also healthy subjects [35], which limits the validity of healthy control groups. Furthermore, the influence of age, sex, axial length, etc. is not sufficiently established to allow for proper selection criteria of a control group [36]. Moreover, since the AMN lesion typically only involves a small fraction of the OCTA scan, it is unclear if the whole scan area or just the area of the AMN lesion should be compared, and if the latter, it remains unclear of how to properly transferring this region onto other eyes with different foveal and vascular architecture. Also for intraindividual comparison with the partner eye, this issue persists. Furthermore, we found no conclusive literature on the correlation of VAD in-between both eyes of the same subject; especially, we found no evidence that VAD should strongly correlate between left and right eye in general and for specific regions within the scan in special.

We therefore used a comparator within the same eye. Since the retinal vascular architecture is designed centripetally towards the fovea, our control parameters ATC and RS were chosen so that they should be mostly homogenous within the same eye. However, while we found significant results for the directly adjacent tissue control, the comparison to the ring segment nearly missed the significance threshold. Previous studies have shown that VAD measurements between different quadrants of the same ring segment can alter substantially [35], which might explain higher fluctuations in ring segment VAD and therefore might make reaching significance threshold more complicated. Nonetheless, also this comparator showed a clear trend towards a reduced choroidal perfusion in AMN.

A third aspect explaining different findings could be that the DCP quantifications are erroneous due to OCT alterations by the AMN. Even though we have argued above that we attribute the alterations rather to the HFL than to the OPL, proper OPL alterations have been reported by many authors; if such exist, they might cause light scattering and reduced OCTA signal. More likely, HFL hyperreflectivity might cause erroneous segmentation of the OPL slab, which consequently could alter VAD measurements especially of the DCP. Even though we acknowledge that the VAD measurements in the choroidal layers might be equally disputable (see next subchapter), this might explain the different findings.

A last consideration regarding the contradicting findings could be the mostly small sample size of the studies (in the studies supporting a DCP origin, only 1 larger study with 16 eyes [12], the rest smaller studies with 3 [11], 2 [10], or 1 eye [3, 8, 13]). All studies supporting a choriocapillary origin of AMN also were based on smaller sample sizes (3 eyes in both [31, 33], 9 eyes in [32]). Moreover, many studies only used descriptive terms that reported of hypoperfusion without proper quantification. Interestingly, all previous studies reporting on a choriocapillary perfusion deficit did not perform proper VAD quantification or statistical analysis. Our work thus is the first to quantify and statistically undermine the theory of a choriocapillary hypoperfusion origin of AMN.

### Susceptibility of choroidal OCTA measurements to artifacts

A non-negotiable factor that limits the findings of the advocates of a choroidal perfusion deficit is the limited usability and accuracy of OCTA for imaging the very. This has been a major factor of dispute over the validity of these findings [9]. A study quantifying choriocapillaris vasculature in case of reticular pseudodrusen found that both larger retinal vessels and pseudodrusen would cause projection artifacts and could alter choriocapillaris quantification measurements [37]. Similarly, AMN-related photoreceptor changes could cause erroneous measurements similar to pseudodrusen. In our study, we found no significant differences in OCTA quantification of the choroidal layers whether areas of large retinal vessels (as identified by the frangi filter in SVP) were excluded in OCTA analysis or not (data not shown). However, the alterations in the photoreceptor layer could be very well argued to cause light scattering and diminished signal from underlying layers in OCTA.

The single B-scans, however, do not show any signs of reduced signal of the choroidal layers underlying the photoreceptor layers (see for example Fig. 4C). In theory, larger vessels can cast a shadow on the underlying retinal layers, whereas RPE atrophy is known to cause relative hyperreflectivity of the underlying choroid (because of missing blockade). In our patients, we found no visible artifact-related fluctuations in choroidal structure in regular OCT. If, however, OCTA might be more susceptible than structural OCT for light scattering artifacts is a question that cannot conclusively be debunked. We do, however, argue that the validity of the reported DCP underperfusion might equally be challenged (see above).

### Interpretation of en face OCT findings

The en face OCT analysis conducted in this study shows that the IR hyporeflectivity correlates better to OCT alterations in the photoreceptor layer rather than the OPL, a finding that has not been reported before. One argument for a perfusion deficit in the DCP is that such would cause swelling of the OPL and thus hyperreflectivity in IR, similar to what can be seen in retinal artery occlusion. Our findings contradict this notion by clearly showing that the IR hyporeflectivity is caused by photoreceptor alterations instead. We believe this to further weaken the DCP perfusion deficit thesis.

### Limitations and strengths of this study

Even though we believe to have put substantial weight on the conjecture of a choroidal perfusion deficit origin of AMN, we agree that also our evidence is not substantial enough to close the case. However, when disregarding the contradictory findings whether DCP or CC perfusion is reduced, we find that the pure theory behind the choroidal origin sounds more plausible than a DCP origin: Why should mainly the photoreceptors be altered in case of a DCP origin, when the photoreceptors are not perfused by it, and why are these alterations not present in the much more severe retinal artery occlusion?

One limitation of this study is the debatable quality of the choroidal OCTA measurements. Another limitation is that we solely discuss perfusion deficits as possible origins of AMN and disregard other thinkable pathomechanisms, e.g., autoimmune processes or other types of inflammation. Also, we only included patients with a SARS-CoV-2 infection into the study. As discussed earlier, SARS-CoV-2 has often been shown to cause AMN [7]; however, we would argue that this is similar to the previously described other viral infections causing AMN. We therefore do not believe that SARS-CoV-2-related AMN is fundamentally different from other AMN, and we found no literature that would indicate otherwise. Thus, we presume that the study’s findings might also be generalized to non-SARS-CoV-2 AMN cases. However, our data cannot conclusively prove this assumption. Likewise, any impact of oral contraceptives on the results of the study cannot be ruled out.

A strength of our study is the unbiased inclusion of all patients in a consecutive case series with proper statistical and not only descriptive analysis. Also, we believe that our approach of releasing our software open source will allow for reproduction studies and/or larger multicenter validation studies.

## Conclusion

Our analysis shows further support of the thesis of a vascular origin of AMN. Within this vascular theory, both our theoretical considerations and our OCT and OCTA analysis favor a choroidal underperfusion rather than the previously hypothesized underperfusion of the DCP. The underlying cause for this underperfusion and the strong epidemiological clustering in young women remains unclear.
